# Molecular mechanisms for environmentally induced and evolutionarily rapid redistribution (plasticity) of meiotic recombination

**DOI:** 10.1093/genetics/iyab212

**Published:** 2021-12-09

**Authors:** Reine U Protacio, Tresor O Mukiza, Mari K Davidson, Wayne P Wahls

**Affiliations:** Department of Biochemistry and Molecular Biology, University of Arkansas for Medical Sciences, Little Rock, AR 72205-7199, USA

**Keywords:** recombination, meiosis, recombination hotspot, plasticity, evolution, Prdm9

## Abstract

It has long been known (circa 1917) that environmental conditions, as well as speciation, can affect dramatically the frequency distribution of Spo11/Rec12-dependent meiotic recombination. Here, by analyzing DNA sequence-dependent meiotic recombination hotspots in the fission yeast *Schizosaccharomyces pombe*, we reveal a molecular basis for these phenomena. The impacts of changing environmental conditions (temperature, nutrients, and osmolarity) on local rates of recombination are mediated directly by DNA site-dependent hotspots (*M26*, *CCAAT*, and *Oligo-C*). This control is exerted through environmental condition-responsive signal transduction networks (involving Atf1, Pcr1, Php2, Php3, Php5, and Rst2). Strikingly, individual hotspots modulate rates of recombination over a very broad dynamic range in response to changing conditions. They can range from being quiescent to being highly proficient at promoting activity of the basal recombination machinery (Spo11/Rec12 complex). Moreover, each different class of hotspot functions as an independently controlled rheostat; a condition that increases the activity of one class can decrease the activity of another class. Together, the independent modulation of recombination rates by each different class of DNA site-dependent hotspots (of which there are many) provides a molecular mechanism for highly dynamic, large-scale changes in the global frequency distribution of meiotic recombination. Because hotspot-activating DNA sites discovered in fission yeast are conserved functionally in other species, this process can also explain the previously enigmatic, Prdm9-independent, evolutionarily rapid changes in hotspot usage between closely related species, subspecies, and isolated populations of the same species.

## Introduction 

In meiosis cells express the broadly conserved Spo11/Rec12 protein which, along with other components of the basal meiotic recombination machinery, catalyzes the formation of double-strand DNA breaks (DSBs) that initiate homologous recombination (Lam and Keeney 2014; Robert *et al.* 2016; Jing *et al.* 2019). The broken chromosome uses its intact homolog as a template for repair, leading to gene conversion (recombination) on the initiating chromosome. A subset of these gene conversion events generate crossovers (reciprocal recombination) between the homologs. As is the case for transcription, meiotic recombination can occur anywhere in the genome, but its distribution is tightly regulated. In diverse species, distribution maps of recombination-initiating DSBs and/or recombination events (*i.e.*, gene conversions, conversion-associated crossovers, or both) revealed that most recombination is tightly clustered at hotspots that regulate the frequency distribution of recombination across the genome (Wahls and Davidson 2012; Choi and Henderson 2015; Dluzewska *et al.* 2018; Ergoren 2018; Jing *et al.* 2019).

The global distribution of DSB hotspots can be defined with precision using several molecular tools (de Massy *et al.* 1995; Wu and Lichten 1995; Baudat and Nicolas 1997; Gerton *et al.* 2000; Blitzblau *et al.* 2007; Buhler *et al.* 2007; Pan *et al.* 2011). The concordance and reproducibility of the different approaches are exemplified by two, extensively studied, highly diverged organisms, fission yeast, and budding yeast. Experiments conducted using different DSB detection methods, at different times, and even in different laboratories have yielded similar patterns for the distribution of DSB hotspots within each of these species, differing primarily in the resolution and sensitivity of the assay employed (Gerton *et al.* 2000; Young *et al.* 2002; Blitzblau *et al.* 2007; Buhler *et al.* 2007; Cromie *et al.* 2007; Pan *et al.* 2011; Fowler *et al.* 2014). However, most of the intra-species comparative studies have used similar genetic backgrounds and experimental conditions, such as the media and temperature in which meiosis was induced. Remarkably, other experiments revealed that differences in metabolic states and environmental cues can trigger dramatic changes in the positioning of recombination at hotspots, even within isogenic or genetically identical strains.

Plasticity in the frequency distribution of meiotic recombination is a long-recognized, well-documented phenomenon (Plough 1917). For example, differences in sex or mating-type affect the distribution of recombination among hotspots of diverse species (*e.g.*, fungi and mammals), as does the addition of an ectopic mating-type cassette (Parvanov *et al.* 2008; Kong *et al.* 2010; Hyppa *et al.* 2014; Brick *et al.* 2018). Both auxotrophies and nutritional states, such as the addition or removal of amino acids in the media, affect hotspot activity (Abdullah and Borts 2001; Cotton *et al.* 2009). Differences in parental mating type and the freezing of diploids each affect the activity of hotspots in subsequent meiosis, which suggests that there is epigenetic imprinting (Parvanov *et al.* 2008; Stahl *et al.* 2016). Such imprinting can also be inferred from the structure and composition of chromatin at hotspots (de Castro *et al.* 2011; Storey *et al.* 2018; Mukiza *et al.* 2019). Differences in temperature during meiosis affect patterns of recombination across the genomes of diverse species (Bomblies *et al.* 2015), and in two of these species the effects of temperature on the global distribution of recombination-initiating DSBs have been examined. In budding yeast only about 20% of DSB hotspots, as defined using a frequency threshold, occur at the same positions when meiosis is carried out at 14°C, 30°C, and 37°C (Zhang *et al.* 2017). In fission yeast differences in temperature affect the global distribution of DSBs (Hyppa *et al.* 2014) and rates of recombination (Brown *et al.* 2020) at the *M26* class of DNA site-dependent hotspots (Kon *et al.* 1997; Steiner and Smith 2005). Hypothetically, such regulatory DNA sites and their binding/activator proteins might contribute to the plasticity of recombination positioning (Zhang *et al.* 2017; Mukiza *et al.* 2019). There are other, even more perplexing manifestations of plasticity. For example, while the transcription factor Prdm9 (which is present in a subset of metazoans) can modulate the initiation of recombination at its own DNA binding sites (Brick *et al.* 2018), the deletion of Prdm9 leads to the generation of new hotspots elsewhere in the genome (Brick *et al.* 2012; Mihola *et al.* 2021). A similar situation applies for transcription factors Bas1 and Ino4 of budding yeast, whose removal represses DSBs at some hotspots and induces DSBs at others (Mieczkowski *et al.* 2006; Zhu and Keeney 2015). In summary, environmental conditions and metabolic states can reshape—in some cases quite dramatically and by yet unknown mechanisms—the frequency distribution of meiotic recombination across the genomes of diverse taxa.

To gain insight into mechanisms for plasticity in the frequency distribution of meiotic recombination, we studied three exceptionally well-characterized classes of recombination hotspots in fission yeast. Each different class of hotspot is regulated by a discrete DNA sequence motif that has been defined functionally at single-nucleotide resolution by systematic, comprehensive, scanning base-pair substitutions in the genome (Table 1; Schuchert *et al.* 1991; Steiner *et al.* 2009, 2011). In each case, the binding of transcription factors to those DNA sites is essential for hotspot activity. The Atf1-Pcr1 heterodimer (of the ATF/CREB/AP-1 family) promotes recombination at *M26* (*CRE*-like) DNA sites (Kon *et al.* 1997; Gao *et al.* 2008); Php2-Php3-Php5 complex activates recombination at *CCAAT* box DNA sites (Steiner *et al.* 2011); and Rst2 regulates recombination at *Oligo-C* DNA sites (Steiner *et al.* 2011). Notably, the removal of these proteins has little or no impact on the rates of recombination for well-matched, basal recombination controls that lack those DNA sites, demonstrating that the binding/activator proteins are hotspot-specific regulators of recombination. These disparate *cis*-acting regulatory modules each share a common downstream mechanism. Each protein-DNA complex triggers the displacement of nucleosomes to promote access of the basal recombination machinery (Spo11/Rec12 complex) to its DNA substrates within chromatin (Mukiza *et al.* 2019), thereby stimulating locally the frequency of recombination-initiating DSBs (Steiner *et al.* 2002; Wahls and Davidson 2010; Fowler *et al.* 2014). Here we reveal mechanisms by which these types of *cis*-acting regulatory modules can reshape the frequency distribution of meiotic recombination across the genome in response to environmental and metabolic cues.

**Table 1:** DNA sequences of *ade6* alleles

*ade6* allele	Relevant sequence^a^
	121 1461
*ade6^+^ (wt)*	AAACAAATTGA.TGGAGGACGTGAGCACAT… AGATGCCTCG
*ade6-M26*	AAACAAATTGA.TGGA**t**GACGTGAGCACAT …AGATGCCTCG
*ade6-CCAAT ^b^*	AAACAAATTGA**t**TGGAGGACGTGAGCACAT …AGATGCCTCG
*ade6-Oligo-C*	t AACAAATTGA **.** **acccc**G**cac** TGAGCACAT …AGATGCCTCG
*ade6-M375*	AAACAAATTGA.T t GAGGACGTGAGCACAT …AGATGCCTCG
*ade6-M210*	AAACAAATTGA.TGGAGGACGTGAGCACAT … AGATGC t TCG

a The *ade6* ORF is 1,659 bp in length and coordinates are numbered relative to the first nucleotide of the start codon (+1). The hotspot (*M26, CCAAT, Oligo-C*), basal recombination control (*M375*), and test-cross (*M210*) alleles differ from wild-type (*wt*) only by the indicated bp substitutions (*lower case*). The bp substitutions of the hotspot alleles create DNA binding sites (*grey shading*) for DNA sequence-specific, hotspot-activating binding proteins (Atf1-Pcr1 heterodimer, Php2-Php3-Php5 complex, and Rst2, respectively). Each allele also renders cells auxotrophic for adenine, allowing one to score for adenine prototrophic recombinants (*ade6^+^*) from heteroallelic crosses that contain a 5’ allele (*M375, M26, CCAAT, or Oligo-C*) and the 3’ allele (*M210*).

b The consensus motif 5’-CCAATCA-3’ is on the complementary strand.

## Materials and methods

### Fission yeast husbandry

The DNA sequences of *ade6* alleles used in this study are provided in Table 1 and the genotypes of all strains are listed in Supplementary Table S1. Strains were constructed using standard genetic techniques and were cultured in rich or minimal media supplemented as necessary with amino acids and bases at 100 µg/ml (Gutz *et al.* 1974; Forsburg and Rhind 2006; Gao *et al.* 2009). Mating and meiosis were carried out on sporulation agar (SPA) that contained 3% agar, 1% glucose, 0.1% K_2_HPO_4_, vitamins and any required supplements. SPA-glu contained 5% glucose and SPA-KCl contained 0.5M KCl. Spores were harvested when asci became abundant, which takes 3–4 days at our standard temperature of 25°C and up to 10 days at 15°C. Ectopic expression of *rst2* was from the plasmid pREP3X-Rst2 (Takenaka *et al.* 2018), which uses the regulatable *nmt1* promoter (Maundrell 1993); thiamine (0.5 µg/ml) was included in the media to maintain low expression levels (Forsburg 1993).

### Measurements of meiotic recombination

Methods to determine rates of meiotic recombination were as described (Kon *et al.* 1997, 1998). In brief, haploid strains with different *ade6* alleles (Table 1) were mated; then, haploid meiotic products (spores) were harvested and serial dilutions of spores were plated on minimal media that contained or lacked adenine. The titer of Ade^+^ recombinant spore colonies was divided by the titer of all viable spore colonies to yield the recombinant frequency of each cross. Frequencies reported in the figures are mean ± SD of values from three or more independent biological replicates of each cross.

### Measurements of mRNA abundance

Quantitative, real-time, reverse-transcription PCR (qRT-PCR) was used to measure the abundance of mRNAs in cells (A_595_ = 0.5) that had been starved for nitrogen for 60 min to induce sexual differentiation. For each sample, total RNA from approximately 1 × 10^8^ cells was extracted using the Quick-RNA Fungal/Bacterial Miniprep (Zymo Research, Irvine, CA, USA). For each sample, 1 µg of RNA was treated with TURBO DNase using the TURBO DNA-*free*^™^ Kit, which includes reagents for the digestion of DNA along with the removal of the enzyme and divalent cations post-digestion. Following DNase treatment, cDNA was synthesized from 400 ng RNA as template using the iScript cDNA Synthesis kit (BioRad, Hercules, CA, USA). The cDNA was used as template for qPCR using BlazeTaq SYBR Green qPCR Master Mix 2.0 (GeneCopeia, Rockville, MD, USA) and the PCR primers listed in Supplementary Table S2; reactions were carried out using a CFX96 Real Time System (BioRad, Hercules, CA, USA). Each qPCR reaction (10 µl) contained 1.0 µl of template and 200 nM of forward and reverse primers. Thermocycler parameters were: one cycle at 95°C for 10 min; followed by 40 cycles of 95°C for 10 s, 60°C for 20 s, and 72°C for 15 s. In each experiment, specificity was confirmed by melting point analyses from 65°C to 95°C at 0.5°C increments. For each transcript and experimental condition, fold change in mutant *vs* wild-type samples was calculated using the ΔΔCt method normalized to *cam1* as the internal control (Livak and Schmittgen 2001; Schmittgen and Livak 2008). Calculations used the equation: ΔΔCt = [(Ct gene—Ct ref) in wild-type]—[(Ct gene—Ct ref) in mutant], where gene refers to transcript being measured (*atf1, pcr1, php2, php3, php5*, and *rst2*), and ref is the *cam1* reference transcript. Values reported in the figures are mean ± SD of values from four independent biological replicates.

### Measurements of protein-DNA binding

Reagents, methods and controls for chromatin immunoprecipitation (ChIP) of epitope-tagged Pcr1 (Pcr1-HA) were as previously described (Ekwall and Partridge 1999). Analyses included vegetative cells in log-phase growth and cells in meiotic prophase (Mukiza *et al.* 2019). Abundance of DNA from ChIP samples was determined by using quantitative PCR with the primers listed in Supplementary Table S2; relative enrichment within each sample (% ChIP signal *vs* input efficiency) was calculated by normalization to DNA abundance in aliquots of the same sample prior to immunoprecipitation. Values reported in the figures are mean ± SD of values from three independent biological replicates.

### Statistics

Each experiment was conducted using three or more independent biological replicates. Two-sided *t*-test was used to calculate significance of differences between recombinant frequencies; linear least squares regression was used for population-level data on dose responses; 95% confidence intervals were used for gene expression values from the ΔΔCt approach. Differences with *P ≤ *0.05 were judged to be significant statistically.

## Results

All known DNA site-specific, recombination hotspot-binding/activating proteins of fission yeast (Atf1, Pcr1, Php2, Php3, Php5, and Rst2) are also transcription factors (Kon *et al.* 1997; Steiner *et al.* 2011). In their roles as environmental condition/stress-responsive transcription factors, each *cis*-acting regulatory module (protein-DNA complex) responds to positive and negative signals to control the rate of transcription of its target genes (*e.g.*, Higuchi *et al.* 2002; Davidson *et al.* 2004; Mercier *et al.* 2008). Hypothetically, such signals could also modulate the rate of recombination at each class of DNA site-dependent hotspots. To test this hypothesis, we compared the effects of environmental and metabolic conditions on recombination at the DNA site-dependent hotspots. We included a well-matched basal recombination control (which lacks any hotspot-activating DNA sequence motif) to distinguish between hotspot-specific and nonspecific modes of regulation. To avoid any changes in the overall structure or spacing of elements in the genome, each allele used in this study was created by only one or a few base pair substitutions within the *ade6* test locus (Table 1).

### The *M26*, *CCAAT*, and *Oligo-C* DNA sites activate recombination hotspots under standard conditions

**Figure 1:**
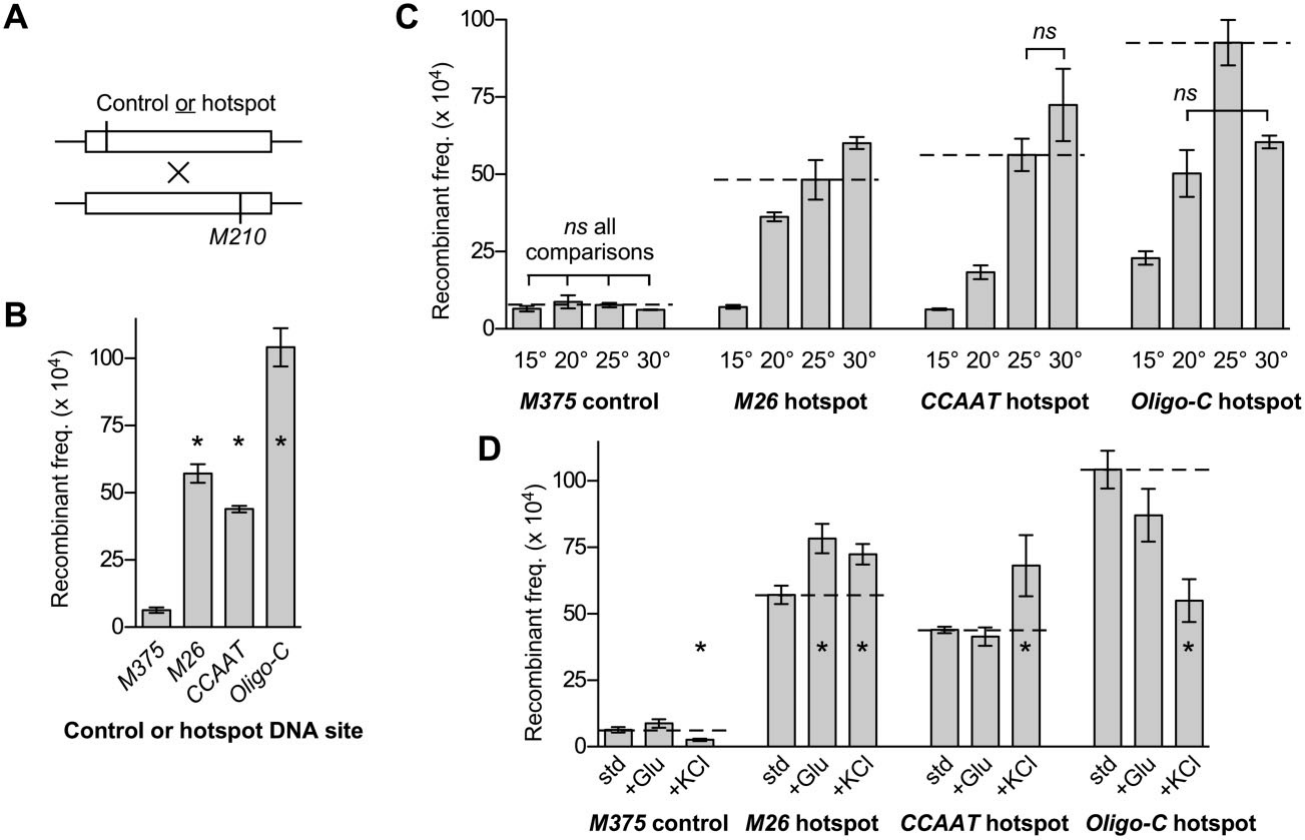
DNA site-dependent recombination hotspots directly modulate rates of recombination in response to changing environmental conditions. (A) Diagram of genetic crosses showing relative positions of *ade6* alleles (see Table  1 for sequences of alleles). (B) Rates of recombination under standard conditions for crosses with hotspot-activating DNA sequence motifs (*M26*, *CCAAT*, and *Oligo-C*) are compared to those of a basal recombination control (*M375*). (C) Effects of temperature (15°C, 20°C, 25°C, and 30°C) on recombination rates. Here and subsequently, rates under standard conditions are depicted for comparison (*horizontal dashed lines*). (D) Effects of increased carbon source (*+Glu*, 5% glucose) and hyperosmolarity (*+KCl*, 0.5 M) on rates are compared to those under standard conditions (*std*, 1% glucose and 0.0 M KCl). Data are mean ± SD from three independent biological replicates. In B and D, differences with *P ≤ *0.05 are indicated (*) for hotspot *vs* control (B) and altered environmental conditions *vs* standard conditions (D). In C, all six possible pairwise comparisons within each data set were significantly different except for those indicated (*ns*, not significant).

We compared the rates of meiotic recombination from crosses of strains bearing either a basal recombination control allele (*M375*) or one of three different hotspot DNA sequence motifs (*M26*, *CCAAT*, and *Oligo-C*) located near the 5′ end of the *ade6* gene (Table 1). Haploid strains with these alleles, which are auxotrophic for adenine, were crossed to another adenine auxotroph that harbors a tester allele (*M210*) near the 3′ end of *ade6* (Figure 1A). After mating and meiosis, haploid spores were plated and the spore colonies were scored for the frequency of Ade^+^ recombinants. Because meiosis is a single round event and the reversion rates of the alleles are negligible, the recombinant frequency provides a measure of recombination rate. In each case, the hotspot DNA sequence motifs (*M26*, *CCAAT*, and *Oligo-C*) promoted the rate of recombination substantially (by about 7-fold to 16-fold), relative to that of the basal recombination control (*M375*) (Figure 1B).

These experiments were conducted using standard, widely employed conditions for mating and meiosis, which are incubation on SPA at 25°C until asci are abundant. We then changed one environmental variable at a time to determine their effects on the specific activities of the recombination hotspots. To facilitate visual comparisons of data in subsequent figure panels, values observed under standard conditions are shown using a horizontal, dashed line.

### Effects of environmental conditions on local recombination rates are mediated directly and differentially by each class of hotspot

Changes in the environment that are encountered regularly by free-living, unicellular organisms (such as fission yeast) include those of temperature, energy source, and osmolarity. We therefore included each of these factors as variables in our experiments.

#### Effects of temperature

We varied the temperature of mating and meiosis, from 15°C to 30°C in 5°C increments, for crosses harboring the hotspot (*M26*, *CCAAT*, or *Oligo-C*) and basal recombination control (*M375*) alleles. For the basal recombination control, changing the temperature had no statistically significant impacts on recombination rates (Figure 1C). These findings indicate that the basal meiotic recombination machinery (Spo11/Rec12 complex) is proficient for initiating recombination in the face of, and is not affected appreciably by, changes in temperature from 15°C to 30°C. In sharp contrast, changing the temperature of meiosis triggered substantial changes in the rates of recombination for each of the DNA site-dependent hotspots (Figure 1C).

For the *M26* hotspot, the differences in the temperature of meiosis led to an approximately 8.6-fold range in rates of recombination (Figure 1C). The rates were lowest at the coolest temperature (15°C) and, relative to that temperature, rose with sequential increases in temperature by about 5.2-fold (20°C), 6.9-fold (25°C), and 8.6-fold (30°C). Each of the differences between the six pair-wise comparisons was statistically significant. Because temperature had no significant impact on rates of recombination for the basal recombination control, we conclude that signals from environmental temperature are transduced though this DNA site-dependent hotspot to control local rates of recombination. We also conclude that individual DNA site-dependent hotspots can modulate recombination rates over a broad dynamic range.

For the *CCAAT* hotspot, varying the temperature led to an approximately 11-fold range in the rates of recombination (Figure 1C). Recombination was lowest at the coolest temperature (15°C) and, relative to that value, was stimulated at higher temperatures by about 2.9-fold (20°C), 8.9-fold (25°C), and 11-fold (30 C). Five of the six inter-sample differences were significant. We conclude that, like the *M26* hotspot, the *CCAAT* hotspot controls local rates of recombination over a broad dynamic range in response to changes in temperature.

For the *Oligo-C* hotspot, the changes in temperature led to an approximately 4.1-fold range in recombination rates (Figure 1C). There was a statistically significant, biphasic response where the rates increased in the 15°C to 25°C range, then decreased between 25°C and 30°C. Thus, like the *M26* and *CCAAT* hotspots, the *Oligo-C* hotspot directly mediates environmentally induced changes in local recombination rates. Like the other hotspots, this hotspot functions as a rheostat that modulates the activity of the basal recombination machinery. The lower dynamic range for control of recombination by this *Oligo-C* hotspot, relative to the others, is attributable to the fact that this hotspot retained substantial activity at the 15°C temperature, whereas the *M26* and *CCAAT* hotspots behaved similarly to the basal control *M375*.

Although each class of DNA site-dependent recombination hotspots responded to changes in temperature, there were clear differences between the hotspots. There were statistically significant differences in minimum hotspot activity, maximum activity, dynamic range, optimal temperature, and temperature response profiles (Figure 1C). For example, at 15°C the *Oligo-C* hotspot promoted recombination substantially (3.5-fold higher recombination rate than for the basal recombination control *M375*), whereas the *M26* and *CCAAT* hotspots did not (*i.e.*, had rates equivalent to that of the basal recombination control). As another example, the *Oligo-C* hotspot had maximal activity at 25°C, whereas the *M26* and *CCAAT* hotspots were most active at 30°C. Such differences in the data set revealed unambiguously that individual, discrete, seemingly modest changes in the environment can elicit substantially different (even opposite) dynamic responses among different classes of hotspots. For example, changing the temperature from 25°C to 30°C increased significantly the activity of the *M26* hotspot while significantly reducing the activity of the *Oligo-C* hotspot. We conclude that each class of DNA site-dependent hotspots can respond independently and differentially, relative to other classes of hotspots, to the same environmental cues.

#### Effects of energy source

Our standard SPA contains 1% glucose as the energy source. We compared rates of recombination under these conditions to rates under conditions of elevated glucose (5%) (Figure 1D). This increase in glucose had no significant impacts on recombination rates for the basal recombination control. However, rates of recombination for the *M26* hotspot were increased significantly, those for the *CCAAT* hotspot were unchanged, and there was a nonsignificant decrease for the *Oligo-C* hotspot. We conclude that signals from the environmental abundance of this key nutrient are transduced through at least one of these DNA site-dependent hotspots to control local rates of recombination. We do not mean to imply that the other hotspots are insensitive to carbon source availability; they might be affected by glucose concentrations or energy sources not tested in this study. Notwithstanding this possibility, the observed differential responses further support the idea that each class of hotspots can be regulated independently by its own constellation of signals.

#### Effects of osmolarity

Our standard SPA formulation contains no osmolytes or salts other than the 7 mM potassium phosphate buffer. As the variable, we supplemented the SPA with 500 mM KCl (Figure 1D). For the basal recombination control (*M375*), this increase in osmolarity led to a significant decrease in the rates of recombination. In contrast, there were significant increases in recombination rates for the *M26* and *CCAAT* hotspots, as well as a significant reduction in rates for *Oligo-C*. Thus, as for changes in temperature and glucose, different classes of DNA site-dependent hotspots respond differentially to changes in osmolarity. However, in this case (osmolarity), the magnitude of the hotspot-specific effects is partially obscured by the fact that KCl also triggered a reduction in basal recombination. In such cases, the hotspot-specific component of control can be ascertained by comparing hotspot activity ratios, which are the rates of recombination for a given hotspot under a given condition divided by the rates for the basal recombination control under the same conditions. By these criteria (using *M375* control-normalized data), the hotspot-specific impacts of adding KCl were about 3.1-, 3.6-, and 1.3-fold increases in recombination, respectively, for the *M26*, *CCAAT*, and *Oligo-C* hotspots. Both ways of considering the data, either by measuring absolute levels of recombination or by taking into account the hotspot-independent impacts on KCl on basal recombination, support the same conclusions: Signals from changes in osmolarity, like those from changes in temperature and nutrients, are transduced through DNA site-dependent hotspots to help control local rates of recombination. Moreover, as seen for the other environmental cues, the impacts of osmolarity on local rates of recombination are mediated differentially by distinct classes of hotspots.

#### Key conclusions and overarching model for the plasticity of meiotic recombination

Together these results support three fundamental conclusions. First, the impacts of environmental conditions on local rates of meiotic recombination are mediated directly and primarily by DNA site-dependent recombination hotspots. Second, individual DNA site-dependent hotspots can modulate rates of recombination over a very broad dynamic range—even in the same, genetically identical strain. They can, in response to changing conditions, range from not promoting recombination beyond basal levels to being highly active at promoting local functions of the basal meiotic recombination machinery (Spo11/Rec12 complex). Third, different classes of DNA site-dependent hotspots respond independently and differentially, relative to other classes of hotspots, to environmental cues. For example, discrete changes in the environment that increase the activity of one class of hotspots can decrease the activity of another class of hotspots. These striking discoveries revealed a molecular basis for the environmentally induced plasticity of meiotic recombination. Importantly, the independent modulation of recombination rates over a broad dynamic range by each different class of hotspots can trigger substantial, highly dynamic changes in the global frequency distribution of recombination (see model in Figure 7 of the Discussion section).

### Components of environmental condition-responsive signaling pathways control the rate of recombination at each class of hotspots

The impacts of environmental conditions on hotspot activity (Figure 1) must be transduced by intracellular signaling pathways. In support of this idea, the activation of *M26*-class hotspots is affected positively and negatively by multiple different signaling pathways, including those that respond to environmental and metabolic cues (Kon *et al.* 1998; Mizuno *et al.* 2001; Hirota *et al.* 2003, 2004, 2007, 2008; Yamada *et al.* 2004; Gao *et al.* 2009; Storey *et al.* 2018). Notably, each of the hotspot-binding/activating proteins (Atf1, Pcr1, Php2, Php3, Php5, and Rst2) is also a transcription factor that can affect the expression of target genes directly via binding to their promoters and indirectly via inter-pathway connections (*e.g.*, Davidson *et al.* 2004). This raises an interesting question with regard to the activation of meiotic recombination hotspots. Do the hotspot-activating proteins control exclusively hotspot activity at their own DNA sites, or can they also help to control DNA site-dependent hotspots to which they do not bind? To address this question, we analyzed rates of recombination in null mutants lacking each of the hotspot-binding/activating proteins, and we included all possible pairwise combinations of the activator proteins and DNA sequence motifs.

#### Direct control of hotspots

**Figure 2:**
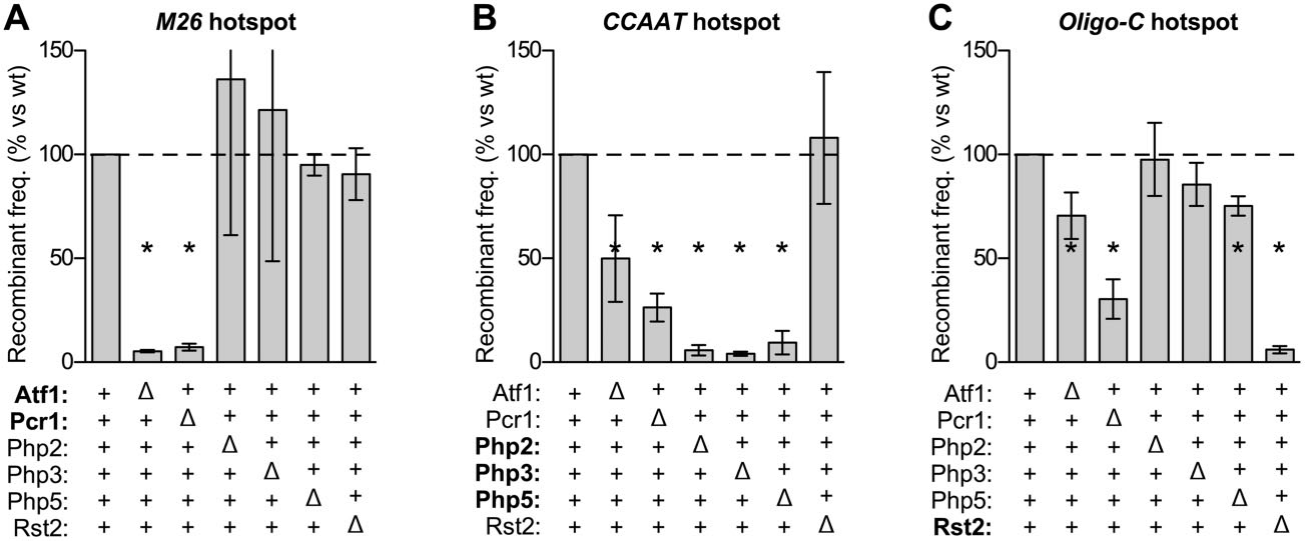
Components of environmental condition-responsive signaling pathways control DNA site-dependent recombination hotspots in *cis* and in *trans*. The effects of each indicated protein on the activation of each class hotspots was measured; proteins that bind directly to each hotspot DNA site are highlighted (*bold*). Relative rates of recombination in null mutant *vs* wild-type are grouped by class of hotspot: (A) *M26* DNA site; (B) *CCAAT* DNA site; (C) *Oligo-C* DNA site. Data are mean ± SD from at least three independent biological replicates; differences with *P ≤ *0.05 (*) are indicated for mutant *vs* wild-type.

Each of the hotspot DNA sequence motifs (*M26*, *CCAAT*, and *Oligo-C*) promoted substantially the rate of recombination, relative to a basal recombination control that lacks those DNA sites (Figure 1B). Removing either subunit of the Atf1-Pcr1 heterodimer, which binds to the *M26* DNA site, greatly reduced the rate of recombination at the *M26* hotspot (Figure 2A). The amount of recombination that remained was similar to that of the basal recombination control allele in wild-type cells. Likewise, subunits of the Php2-Php3-Php5 (*CCAAT* box-binding) complex were required for *CCAAT* motif-promoted recombination (Figure 2B) and Rst2 was required for hotspot activity of its DNA binding site, *Oligo-C* (Figure 2C). These results support a specific conclusion that is germane to the topic of this study: Known components of environmental condition/stress-responsive signal transduction pathways (transcription factors Atf1, Pcr1, Php2, Php3, Php5, and Rst2) control local rates of recombination directly via their own DNA binding sites. We next asked if these proteins could also help to regulate in *trans* other classes of DNA site-dependent hotspots.

#### Indirect control of hotspots

The removal of the Php2, Php3, or Php5 proteins (which bind to and promote recombination at the *CCAAT* motif) had no significant impact on rates of recombination at the *M26* hotspot (Figure 2A). Similarly, the deletions of Php2 and Php3 did not affect appreciably the activation of the *Oligo-C* hotspot (Figure 2C). Likewise, the removal of the Rst2 protein (which binds to and promotes recombination at the *Oligo-C* motif) had no significant impact on the rates of recombination at the *M26* hotspot (Figure 2A) or at the *CCAAT* hotspot (Figure 2B). These findings suggest that the Php2, Php3, and Rst2 proteins function with high specificity to promote recombination at their own DNA binding sites, although it is possible that these proteins help to control other, distinct classes of DNA site-dependent hotspots not examined in this study.

The binding of Atf1-Pcr1 heterodimer to *M26* DNA sites directly activates this class of hotspots. Strikingly, the removal of Pcr1 strongly reduced (by about 74% and 70%, respectively) rates of recombination at the *CCAAT* and *Oligo-C* hotspots (Figure 2, B and C). Removing Atf1 also reduced recombination (by 46% and 30%, respectively) for *CCAAT* and *Oligo-C* (Figure 2, B and C). Similarly, the removal of Php5 significant reduced the activity of the *Oligo-C* hotspot (Figure 2C). We conclude that hotspot-activating transcription factors, which are also key components of environmental condition/stress-responsive regulatory networks, can control the activation of heterologous DNA sequence-dependent hotspots to which they do not bind. The possibility that these *trans* effects are mediated by the indirect recruitment of the activator proteins is explored in greater detail below.

### Hotspot-activating proteins are rate-limiting for promoting, and thus can modulate, recombination at their own DNA binding sites

Results described in the previous two sections revealed that extracellular cues (Figure 1) and components of intracellular signal transduction networks (Figure 2) each modulate the activity of DNA site-dependent recombination hotspots. For this to occur, the *cis*-acting regulatory modules (protein-DNA complexes) must serve as variable-output effectors of those signals. A key prediction of this hypothesis is that each class of DNA sequence motif-dependent hotspots should be sensitive to the abundance of their binding/activating proteins, which we tested as follows.

**Figure 3:**
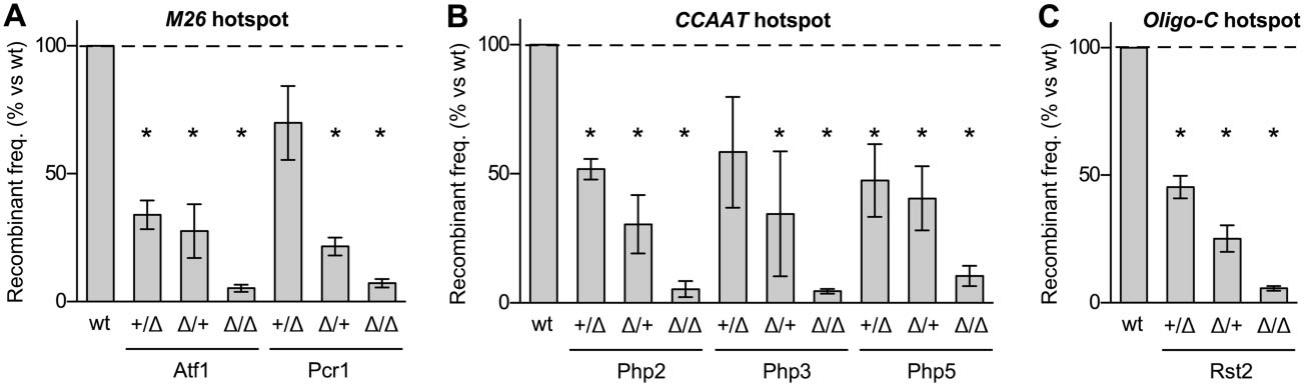
Hotspot-binding/activating proteins are rate-limiting for promoting recombination at their own DNA binding sites. Recombination was measured in crosses that were homozygous wild-type, heterozygous wild-type/null mutant, and homozygous null mutant for hotspot-activating proteins. The heterozygous crosses were in two configurations. In the first configuration (+/Δ), the parent with the hotspot allele was wild-type for the binding protein (*e.g.*, genotype *ade6-M26 atf1^+^*) and the parent with the test-cross allele was null mutant for the binding protein (*e.g.*, *ade6-M210 atf1*Δ). In the second configuration (Δ/+), the parent with the hotspot allele was null mutant for the binding protein (*e.g.*, *ade6-M26 atf1*Δ) and the parent with the test-cross allele expressed the binding protein (*e.g.*, *ade6-M210 atf1*). Rates of recombination are plotted relative to those in homozygous wild-type and are grouped by class of hotspot: (A) *M26* DNA site; (B) *CCAAT* DNA site; (C) *Oligo-C* DNA site. Data are mean from three or more independent biological replicates; differences with *P ≤ *0.05 are indicated for mutant *vs* wild-type (*).

To test for dose-dependent responses, we compared rates of recombination for each hotspot DNA sequence motif (*M26*, *CCAAT*, and *Oligo-C*) in meioses that were homozygous wild-type, heterozygous wild-type/null mutant, and homozygous null mutant for their respective binding proteins. Essentially identical results were observed for each of the six different hotspot-activating proteins (Atf1, Pcr1, Php2, Php3, Php5, and Rst2). In every case, the rate of recombination in the heterozygotes was intermediate between that of homozygous wild-type (full hotspot activity) and homozygous null mutant (no hotspot activity) (Figure 3). Linear regression analysis of the entire data set revealed a robust, positive correlation between dose and recombination rate (*R*^2^ = 0.89, *P < *0.0001). We confirmed this dose-dependent response using second experimental approach that alters the expression of genes without changing their copy numbers (results presented in the next section). The dose-dependent responses, observed using two different approaches, support an important conclusion: Each hotspot-activating protein is rate-limiting for promoting recombination at its DNA binding site. Consequently, any factor (cellular or environmental) that affects the abundance or functionality of a particular, DNA sequence-specific, hotspot-binding/activating protein will affect rates of recombination at the corresponding class of DNA site-dependent hotspots in the genome.

### The control of hotspots is mediated in part by altering expression of the rate-limiting binding/activator proteins

The Atf1 and Pcr1 proteins, which are environmental condition/stress-responsive transcription factors, each controlled the activity of DNA sequence-dependent hotspots that do not contain in their vicinity any binding sites for Atf1-Pcr1 (Figure 2). For example, the removal of Pcr1 reduced by 74% and 70%, respectively, rates of recombination at the *CCAAT* and *Oligo-C* hotspots. Hypothetically, such control could be mediated in *trans* by altering the expression or activity of corresponding binding/activator proteins. Alternatively, the *M26* hotspot-binding/activating protein complex (Atf1-Pcr1 heterodimer) could be recruited by the heterologous hotspots and help to control them in *cis*. We therefore sought to distinguish between these modes of regulation.

#### Atf1-dependent, Pcr1-dependent control of the CCAAT and Oligo-C hotspots is not mediated by the recruitment of Atf1-Pcr1

**Figure 4:**
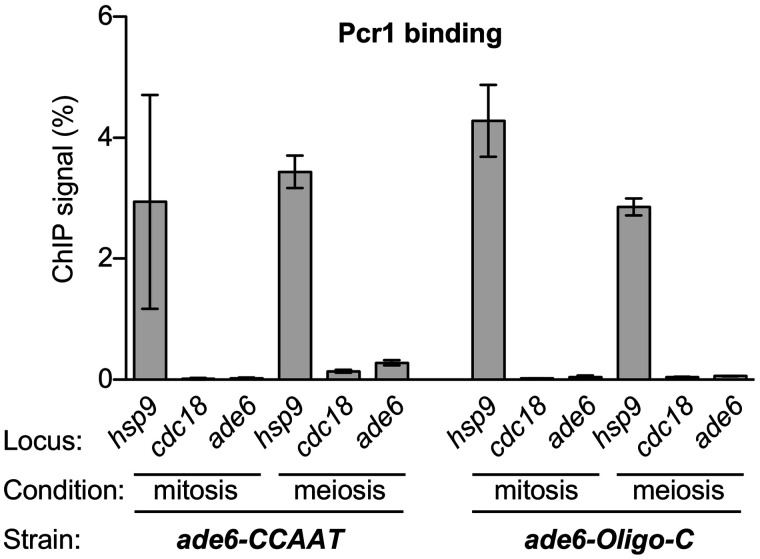
Pcr1-dependent control of the *CCAAT* and *Oligo-C* hotspots is not mediated by the recruitment of Pcr1. Chromatin immunoprecipitation of Pcr1 was used to test whether it is recruited to the test locus (*ade6*) by the *CCAAT* and *Oligo-C* hotspots in vegetative cells (*mitosis*) and cells in meiotic prophase (*meiosis*). Signal intensities from ChIP *vs* the input material used for ChIP (*ChIP signal %*) were compared to those of internal positive (*hsp9*) and negative (*cdc18*) controls. Data are mean ± SD from three independent biological replicates.

ChIP of the Atf1-Pcr1 heterodimer revealed that it binds specifically at *M26* DNA sequence motifs, but not at appreciable levels to our *ade6* test locus in the absence of this DNA site (Eshaghi *et al.* 2010). We used the same approach to test whether presence of the *CCAAT* and *Oligo-C* DNA sites at *ade6* can recruit Pcr1. As an internal, previously validated, positive control, we gauged binding at the *hsp9* promoter; as an internal negative control, we used the *cdc18* ORF (Sanso *et al.* 2008). We analyzed, and obtained equivalent results for, protein-DNA binding in vegetative cells and cells in meiotic prophase (Figure 4). There was about a 25-fold higher ChIP signal for the binding of Pcr1 to the positive control *hsp9* than to the negative control *cdc18*. Under these conditions, we detected no appreciable binding of Pcr1 to either the *CCAAT* or the *Oligo-C* hotspots. We therefore reject the hypothesis that the recruitment of Pcr1 or the Atf1-Pcr1 heterodimer to these hotspots contributes substantially to their activation.

#### Control of the CCAAT and Oligo-C hotspots is mediated in part by regulating expression of their binding/activator proteins

**Figure 5:**
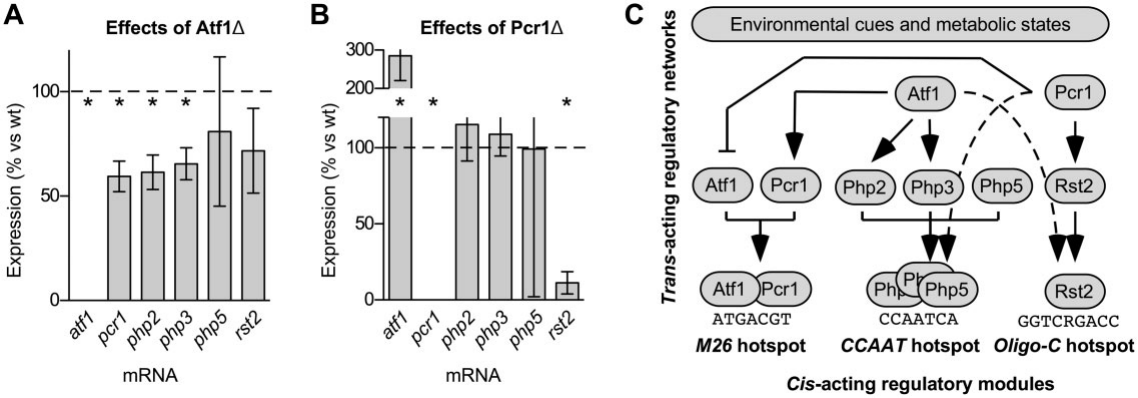
Hotspot-activating proteins can regulate the expression of other hotspot-activating proteins. Quantitative, reverse-transcription PCR was used to determine the effects of Atf1 and Pcr1 on the expression of the six genes (*atf1*, *pcr1*, *php2*, *php3*, *php5*, and *rst2*) that encode hotspot-binding/activating proteins. Gene expression levels in mutants are plotted as % relative to wild-type for: (A) *atf1*Δ mutant; (B) *pcr1*Δ mutant. Data are mean ± SD from four independent biological replicates; differences with *P ≤ *0.05 are indicated for mutant *vs* wild-type (*). (C) Conclusions from this and prior figures. The impacts of environmental conditions on meiotic recombination are mediated via *cis*-acting regulatory modules (site-specific protein-DNA complexes). Transcriptional regulation of hotspot-binding/activating proteins and cross-talk between regulatory networks each contribute to the control of hotspots. Arrows depict transcription-mediated pathway connections elucidated in this study (*solid lines* in upper half of panel), as well as connections where hotspot control in *trans* was also observed, but where there were no significant changes in expression of the genes encoding the respective activator proteins (*dotted lines*).

We next tested whether Atf1 and Pcr1 regulate the expression of genes encoding the proteins that bind to and activate the *CCAAT* box hotspot (Php2-Php3-Php5 complex) and the *Oligo-C* hotspot (Rst2). We used conditions for inducing sexual differentiation that are known to trigger Atf1-Pcr1 heterodimer-dependent changes in the expression of both its direct and indirect targets, such as *cgs2* and *ste11* (Davidson *et al.* 2004). Quantitative, real-time, reverse-transcription PCR (qRT-PCR) was used to compare the relative abundance of each mRNA in null mutant cells *vs* wild-type (Figure 5). It was reported previously, based on quantitative Northern blot analyses of gene expression under conditions similar to those used in this study, that Atf1 promotes the expression of *pcr1* and that Pcr1 represses the expression of *atf1* (Kon *et al.* 1998). We obtained concordant results using qRT-PCR: removing Atf1 reduced the expression of *pcr1* by 40% (1.7-fold difference) (Figure 5A) and ablating Pcr1 increased by 2.9-fold the expression of *atf1* (Figure 5B).

Overall, considering all six genes analyzed, the removal of Atf1 reduced significantly the expression of *pcr1*, *php2*, and *php3* (Figure 5A); whereas the removal of Pcr1 significantly increased the expression of *atf1* and reduced that of *rst2* (Figure 5B). We conclude that some hotspot-activating proteins (which are also transcription factors) can regulate the expression of other hotspot-activating proteins. Our finding that deletions of *atf1* and *pcr1* affected differentially the expression of the other genes was not surprising, given that Atf1 and Pcr1 (like bZIP proteins in general) can form combinatorial homodimers and heterodimers, each of which regulates the expression of a different set of genes (Wahls and Smith 1994; Kon *et al.* 1998; Sanso *et al.* 2008; Eshaghi *et al.* 2010).

The regulated expression of genes encoding hotspot-binding/activating proteins, coupled with the finding that those activator proteins are rate-limiting for promoting recombination at their own DNA sites (Figure 3), provided insight into mechanisms by which hotspots can be controlled in *trans*. The removal of Pcr1 led to an 84% reduction in the expression of *rst2* (Figure 5B) and, correspondingly, reduced by 70% the rate of recombination at the *Oligo-C* hotspot (Figure 2C), which is activated by the binding of Rst2. Similarly, the removal of Atf1 led to reductions in the expression of *php2*, *php3*, and *php5* (reduced by 39%, 35%, and 19%, respectively) (Figure 5A). This led, correspondingly, to a 46% reduction in the rate of recombination at the *CCAAT* hotspot (Figure 2B), which is activated by the binding of the Php3-Php3-Php5 complex. These findings indicate that the regulation of the *Oligo-C* and *CCAAT* hotspots are each mediated, at least in part, via the transcriptional control of the respective hotspot-binding/activating proteins (*pcr1* → *rst2* → recombination at *Oligo-C* hotspot; *atf1* → *php2, php3* → recombination at *CCAAT* hotspot; see pathway diagram in Figure 5C). This type of signal transduction mechanism, exerted through modulating the expression of rate-limiting activator proteins, has broad implications for how diverse signaling networks can affect dynamically the frequency distribution of recombination across the genome. We do not mean to imply that this is the sole or even the primary mechanism for controlling hotspot activity; additional, transcription-independent modes of control could also impinge upon the rate-limiting, hotspot-binding/activating proteins.

#### Deficient activation of the Oligo-C hotspot in pcr1Δ cells is complemented by ectopic expression of rst2

**Figure 6:**
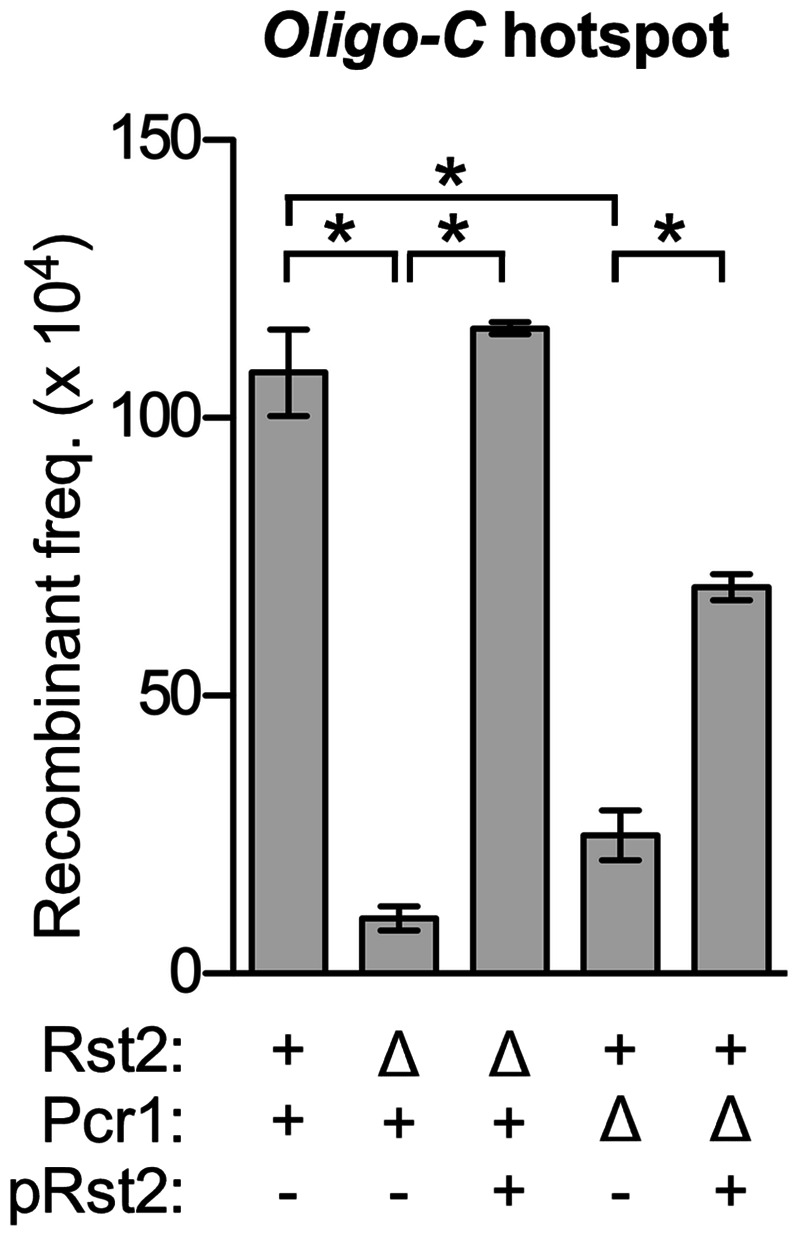
Ectopic expression of *rst2* from a heterologous promoter complements deficient activation of the *Oligo-C* hotspot in *pcr1*Δ cells. Plot shows rates of recombination for the *Oligo-C* hotspot in the presence and absence of its binding/activating protein (*Rst2*), an environmental condition-responsive transcription factor that does not bind to the *Oligo-C* hotspot (*Pcr1*), and a plasmid that expresses *rst2* ectopically from a heterologous promoter (*pRst2*). Data are mean ± SD from three independent biological replicates; relevant significant differences are indicated (*).

Our conclusion that there are transcription-based pathway mechanisms for the control of meiotic recombination hotspots (*e.g.*, *pcr1* → *rst2* → recombination at *Oligo-C* hotspot) stemmed from—and required—experiments in which the regulatory transcription factors were deleted. However, deleting transcription factors can have pleiotropic effects. We therefore sought confirmation that dysregulation of the *rst2* mRNA in *pcr1*Δ mutants (Figure 5B) is a specific factor that compromises activation of the *Oligo-C* hotspot in the in *pcr1*Δ strains (Figure 2C). If so, then adding back some of the missing *rst2* transcript should restore functionality. To test this, we measured rates of recombination at the *Oligo-C* hotspot in the presence and absence of Rst2 (its binding/activator protein), Pcr1, and a plasmid that expresses *rst2* from a heterologous promoter (Figure 6). The removal of Rst2 abolished hotspot activity of its DNA binding site (*Oligo-C*) and ectopic expression of *rst2* from the plasmid restored hotspot activation. Thus, the *rst2* mRNA (and Rst2 protein) expressed from this plasmid is biologically active. The removal of Pcr1 strongly reduced (by about 80%) the rate of recombination at the *Oligo-C* hotspot, and this defect was complemented substantially by the ectopic expression of *rst2*. The ectopic expression of *rst2* did not fully restore recombination to wild-type levels in the *pcr1*Δ strain, suggesting that the deletion of *pcr1* has effects other than downregulation of *rst2* expression, as expected for a transcription factor with several other roles. Nevertheless, the substantial complementation provides independent validation of our conclusion that the control of the *Oligo-C* hotspot in *trans* by Pcr1 is mediated in part by the Pcr1-dependent transcriptional regulation of *rst2*.

#### Evidence for additional, transcription-independent regulatory mechanisms

Interestingly, our qRT-PCR data also revealed that the regulated transcription of genes encoding hotspot-binding/activating proteins cannot be the sole mechanism for regulating hotspots in *trans*. For example, Pcr1 was strongly required for the activation of the *CCAAT* box hotspot: rates of recombination in the *pcr1*Δ mutant were only 26% of those in wild-type (Figure 2B). However, this removal of Pcr1 had no significant impact on the expression of *php2*, *php3*, and *php5* (Figure 5B). The expression levels of these three genes in the *pcr1*Δ mutant were 115%, 109%, and 99% of those in wild-type cells. These findings are inconsistent with a model in which the Pcr1-dependent control of this hotspot is exerted via altering the expression of its binding/activating proteins, Php2, Php3, and Php5. We infer that there must be at least one additional mechanism for the control of DNA site-dependent hotspots in *trans*.

## Discussion

In diverse taxa, environmental cues and metabolic states can reshape dramatically the frequency distribution of meiotic recombination. To explore potential mechanisms for these phenomena, we analyzed the control of recombination by each type of DNA site-dependent recombination hotspot in fission yeast whose binding/activator proteins are known. About 200 additional, distinct DNA sequence elements also activate recombination hotspots in fission yeast, but their binding proteins are unknown (Steiner *et al.* 2009). Processes that modulate the activities of hotspots uncovered in this study likely apply to many, if not most of those other DNA sequence-dependent hotspots. Moreover, because the regulation of hotspots by specific DNA sites and their binding proteins is conserved between species whose latest common ancestor occurred about 400 million years ago (Sipiczki 2000; Steiner and Steiner 2012; Wahls and Davidson 2012) and is implicated by association to be even more widely conserved (*e.g.*, Mougel *et al.* 2014), the mechanisms revealed by this study likely apply broadly across taxa.

### A molecular basis for environmentally and metabolically induced plasticity in the frequency distribution of meiotic recombination

**Figure 7:**
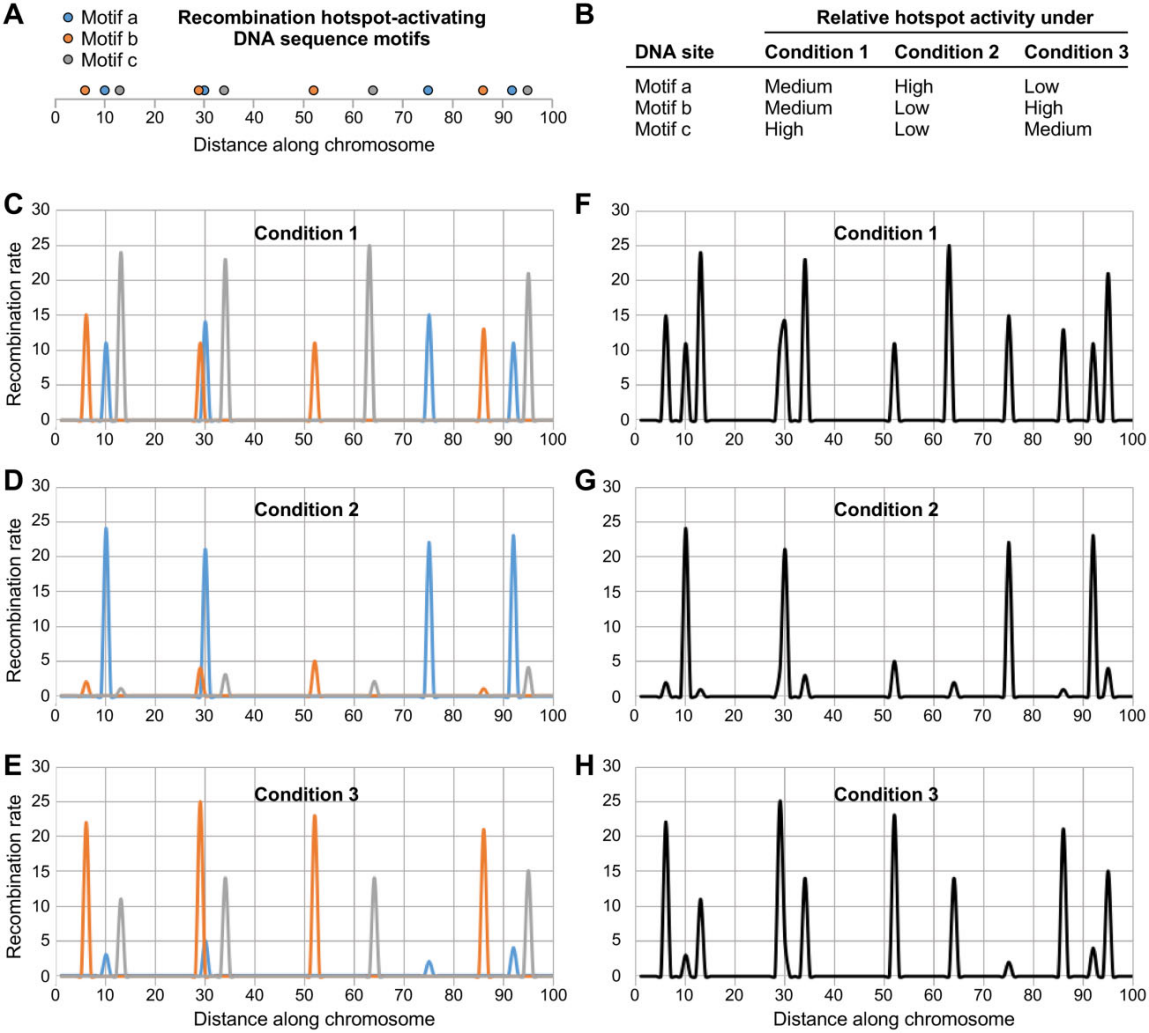
Model for plasticity in the distribution of meiotic recombination. The magnitudes of dynamic changes in relative hotspot activity depicted here are based on those defined experimentally in this study. For the sake of illustration, rates of recombination (arbitrary units) were assigned at random for *low* (rate range of 1–5), *medium* (11–15), and *high* (21–25) activity of individual hotspots. (A) Conceptual distribution of three different types of hotspot-activating DNA sequence motifs (*a*, blue*; b*, orange*; c*, gray) along the chromosome. (B) Each different class of DNA site-dependent hotspots is regulated independently, through its own binding/activating proteins, in response to changes in environmental and metabolic conditions. (C–E) Contribution of each DNA site to the rate of recombination in its vicinity (blue, orange, and gray lines) under the experimental conditions listed in B. (F–H) Net impact of the three classes of regulatory DNA sites on the overall distribution of recombination (*black lines*) under the three different conditions. Note substantial differences in the frequency distribution of recombination events. Also note that if one applies a cutoff value for what is a hotspot (*e.g.*, local recombination rate ≥ 10), as is often done in the literature, the number of annotated hotspots and their apparent positions change substantially from condition to condition.

Our experiments revealed the following. First, the impacts of differences in various environmental conditions (temperature, energy source, and osmolarity) on local rates of recombination are mediated directly and primarily by DNA site-dependent recombination hotspots (Figure 1). Second, and correspondingly, components of intracellular signal transduction pathways that respond to environmental conditions control local rates of recombination through DNA site-specific hotspots (Figures 2 and 3). Third, individual hotspots can modulate rates of recombination over a broad dynamic range—even in the same (*i.e.*, genetically identical) strain (Figure 1). They can, in response to changing conditions, range from not promoting recombination beyond basal levels to being highly active at promoting local functions of the basal recombination machinery. Fourth, relative to each other, different classes of DNA site-dependent hotspots respond differentially to the constellation of environmental cues and metabolic conditions (Figures 1 and 2). For example, discrete changes in the environment that increase the activity of one class of hotspots can reduce the activity of another class of hotspots. These findings revealed—more than a century after environmentally induced plasticity was discovered (Plough 1917)—a molecular basis for dramatic changes in the recombination landscape in response to extracellular and intracellular conditions. Together, the independent modulation of recombination rates by each different class of DNA site-dependent hotspots can remodel dynamically and extensively the distribution of recombination across the genome (see model in Figure 7).

### Multiple mechanisms for controlling recombination rates via hotspot-activating proteins—with implications for complex regulatory networks and the Butterfly Effect

In addition to revealing molecular processes by which extracellular and intracellular conditions can reshape the global frequency distribution of meiotic recombination, our results provide insight into discrete mechanisms that control protein-DNA complex-dependent recombination hotspots.

The first type of mechanism for controlling hotspot activity is exerted through controlling the abundance of the hotspot-binding/activating proteins, which are rate-limiting for promoting recombination at their own DNA sites (Figure 3). For example, removing the environmental condition/stress-responsive transcription factor Pcr1 led to an 84% reduction in the expression of *rst2* (Figure 5B) and, correspondingly, led to a 70–80% reduction in recombination at the *Oligo-C* hotspot (Figures 2B and 6), which is activated by the binding of Rst2. We obtained evidence for the use of this same type of transcription-mediated pathway mechanism for controlling two different classes of hotspots (*pcr1* → *rst2* → recombination at *Oligo-C* hotspot; *atf1* → *php2, php3* → recombination at *CCAAT* hotspot; see pathway diagram in Figure 5C). Moreover, we confirmed that ectopic expression of the dysregulated gene from a heterologous promoter complements the hotspot-activation defect (Figure 6). We note that any other factors (cellular or environmental) whose signals modulate the expression of the rate-limiting, hotspot-binding/activating proteins would also modulate rates of recombination at their DNA sites of action.

A second type of mechanism for controlling hotspot activity can be exerted by changing the functionality of hotspot-binding/activating proteins without necessarily changing their abundance. Our finding that Pcr1 strongly controls activation of the *CCAAT* box hotspot (Figure 2B) without affecting significantly the expression of *php2, php3*, or *php5* (Figure 5B) supports this idea. There are several ways to achieve robust, transcription-independent control of hotspots. For example, signals that affect the subcellular localization, DNA binding affinity, or specific activity of rate-limiting, hotspot-binding/activating proteins will control rates of recombination at their DNA sites of action. An excellent case in point is the hotspot-activating Atf1-Pcr1 heterodimer, whose functions are controlled by each of these three different processes (Gaits *et al.* 1998; Kon *et al.* 1998; Neely and Hoffman 2000; Lawrence *et al.* 2007; Gao *et al.* 2008, 2009).

A third, currently hypothetical type of mechanism for controlling hotspot activity lies in the fact that each of the *cis*-acting regulatory modules analyzed in this study promotes recombination through downstream chromatin remodeling pathways (Mukiza *et al.* 2019). Consequently, any signals that affect chromatin remodelers that help to activate a given class of hotspots would contribute to the regulation of those hotspots. A recent, proteomics-based approach revealed that many different chromatin remodeling enzymes and histone PTMs function in concert to activate individual hotspots (Storey *et al.* 2018), so there are many potential targets for the modulation of hotspot activity at this level.

The multiple different modes of hotspot control are not mutually exclusive and likely operate in concert with each other. Moreover, as with other biological processes, the control of recombination hotspots occurs within the context of, and is likely affected by, complex regulatory networks. Indeed, our discovery that hotspot-binding/activating proteins can help to control the activation of DNA site-dependent hotspots to which they do not bind (Figures 2, 4–6) provides a striking example of how inter-pathway cross-talk can help to shape the positioning of meiotic recombination.

In summary, there are many different classes of DNA sequence-dependent hotspots and multiple different ways to modulate rates of recombination at each class of hotspots via their own, rate-limiting, hotspot-binding/activating proteins. Each of these known and hypothetical mechanisms provides a nexus for the control of hotspot activity by other factors and signaling networks. As in the Butterfly Effect, even minor perturbations to the biological system can, through the diversity of hotspots, inter-pathway connections, and regulatory networks, have large impacts on the overall distribution of recombination (see model in Figure 7).

### A molecular basis for the off-target effects of hotspot-activating proteins in other species

In mice and rats the sequence-specific, hotspot-activating protein Prdm9 represses the activity of some hotspots to which it does not bind (Brick *et al.* 2012; Mihola *et al.* 2021), although it is not yet known whether those other hotspots are controlled by their own DNA sites. Similarly, transcription factors Bas1 and Ino4 of budding yeast affect the frequency distribution of DSBs for hotspots both at their DNA binding sites and elsewhere (Mieczkowski *et al.* 2006; Zhu and Keeney 2015), although those studies did not test for DNA sequence dependence of the presumptively direct or indirect effects. In contrast, this study revealed dependent pathway relationships between multiple different proteins and DNA sites (*e.g.*, Figure 2). Moreover, we identified molecular mechanisms for inter-pathway connections (Figures 4–6). Notably, Prdm9, Bas1, and Ino4 are each transcription factors—as is each currently known, hotspot-binding/activating protein in fission yeast. Thus, the previously enigmatic, off-target effects of Prdm9, Bas1, and Ino4 on the distribution of recombination (Mieczkowski *et al.* 2006; Brick *et al.* 2012; Zhu and Keeney 2015; Mihola *et al.* 2021) are readily explained by our findings and model. Deleting a given hotspot-binding/activating protein (transcription factor) will affect not only the rate of recombination at its own DNA binding sites. It will also affect, via transcription-dependent regulatory networks, all biological processes downstream of that transcription factor. Some of those signals will impinge (positively or negatively) on the binding/activator proteins for other classes of DNA site-dependent recombination hotspots (as documented in Figures 2, 5, and 6), thereby mediating the off-target changes in local recombination rates elsewhere in the genome.

### A molecular basis for the evolutionarily rapid “repositioning” of Prdm9-independent hotspots

A subset of metazoans (*e.g.*, mice, cattle, and primates) express the sequence-specific DNA binding protein Prdm9, whose binding sites activates meiotic recombination hotspots located in intergenic regions (reviewed by Grey *et al.* 2018; Paigen and Petkov 2018). Species that lack Prdm9 or one of its crucial domains (*e.g.*, fungi, birds, amphibians, many fishes, canids, marsupials, and plants) still have hotspots which tend to cluster in a “yeast-like” fashion around functional elements such as promoters (*e.g.*, Munoz-Fuentes *et al.* 2011; Axelsson *et al.* 2012; Kawakami *et al.* 2017). Moreover, Prdm9-expressing species can still activate hotspots that cluster at promoters (remote from Prdm9 binding sites) (Schield *et al.* 2020), as do mice and rats when Prdm9 is deleted (Brick *et al.* 2012; Mihola *et al.* 2021). There seems to be an evolutionarily ancient, Prdm9-independent mechanism for distributing recombination to hotspots. This mechanism likely involves a constellation of hotspot-activating DNA sites like those discovered in fission yeast, the majority of which are known to be conserved functionally between species whose latest common ancestor was about 400 million years ago (Steiner and Steiner 2012; Wahls and Davidson 2012) and that are implicated by association to be even more widely conserved (*e.g.*, Mougel *et al.* 2014).

Prdm9 is among the most rapidly evolving proteins known and the changes in its zinc finger domain change its DNA site specificity, thereby relocating Prdm9-dependent hotspots over short evolutionary time scales. It has been assumed that this property is unique to Prdm9-class hotspots and that the positions of other types of hotspots are more stable evolutionarily, “even over tens of millions of years of evolution” (*e.g.*, Grey *et al.* 2018). However, a growing body of evidence suggests that the positions of Prdm9-independent hotspots can change rapidly over evolutionary time scales. For example, in flycatcher birds there are substantial differences in the positions of annotated hotspots between pairs of closely related species (55–61% of hotspots in different locations) and between isolated populations of the same species (31–49% in different locations) (Kawakami *et al.* 2017). Similarly, about 80% of mapped hotspots are at different locations in subspecies of rice (Marand *et al.* 2019). Likewise, about 85% of inferred hotspots are in different positions in isolated populations of stickleback fish, which became separated from each other within the last 15 thousand years (Shanfelter *et al.* 2019). Such evolutionarily rapid changes in the mapped positions of Prdm9-independent recombination hotspots are perplexing because the low amounts of genomic DNA sequence divergence involved preclude the possibilities of large-scale changes in the DNA sequences of *cis*-acting regulatory elements or in the DNA binding site specificities of the various binding/activator proteins. A solution to this quandary lies in our discovery of mechanisms that can immediately affect local recombination rates, resulting in an overall redistribution of recombination (Figure 7). Importantly, these mechanisms do not require any changes in the distribution of the regulatory DNA sites themselves.

The inference that the positions of hotspots can change rapidly over evolutionary time scales and the implications of such movement have each been influenced strongly by the standard practice of defining recombination hotspots based on either/or cutoff frequency values. However, as shown directly in this study, and as can also be inferred indirectly from data in other studies (Pengelly *et al.* 2016; Kawakami *et al.* 2017; Shanfelter *et al.* 2019), individual, Prdm9-independent hotspots actually function as rheostats that can modulate rates of recombination over a very broad dynamic range (Figure 1). Moreover, as little as a single heterology in the genome can be sufficient to strongly adjust these rheostats, as shown directly by mutating individual components of signal transduction networks (Figure 2). We therefore posit that even minor genetic or metabolic differences between species, subspecies and isolated populations can; by affecting signal transduction networks that differentially control each class of DNA site-dependent hotspots; trigger substantial changes in the distribution of recombination across the genome (as in Figure 7). In short, the dynamic modulation of local recombination rates by different classes of DNA site-dependent hotspots—which can be induced at laboratory/experimental time scales—can explain why Prdm9-independent hotspots (as defined previously using either/or cutoff frequency values) appear to move rapidly over evolutionarily time scales. This postulate has substantial implications for the conservation of fundamental mechanisms that control the distribution of meiotic recombination in diverse taxa and for current thinking about its evolution.

## Conclusions

The striking, previously enigmatic plasticity in the frequency distribution of meiotic recombination across genomes is readily explained by the fact that many, distinct classes of DNA sequence motifs each control rates of recombination locally. Each different type of *cis*-acting regulatory module (hotspot-activating protein-DNA complex) serves as an independently controlled rheostat that modulates rates of recombination over a broad dynamic range in response to environmental cues and metabolic states. Together, the independent modulation of recombination rates by each different class of DNA site-dependent hotspots can remodel dynamically and profoundly the distribution of recombination across the genome (as depicted in Figure 7). This process can also explain why the distribution of recombination among hotspots varies markedly between closely related species, subspecies, and isolated populations of the same species that lack Prdm9.

## Data availability

All data necessary for confirming the conclusions presented in this article are represented fully within the tables and figures. Full genotypes of strains used are provided in Supplementary Table S1; DNA sequences and positional coordinates of the PCR primers employed are provided in Supplementary Table S2. Yeast strains and other materials generated by the study are available upon request.

Supplementary material is available at *GENETICS* online.

## Supplementary Material

iyab212_Supplementary_Data
