# Supplementary material for: Molecular mechanisms for environmentally induced and evolutionarily rapid redistribution (plasticity) of meiotic recombination
Source: Genetics. 2021 Dec 9;220(2):iyab212. doi: 10.1093/genetics/iyab212 (PMC9097252; doi:10.1093/genetics/iyab212)
Supplement: iyab212_Supplementary_Data [file iyab212_Supplementary_Data.pdf]

Supplementary Material

Molecular mechanisms for environmentally induced and evolutionarily rapid redistribution (plasticity) of meiotic recombination

Reine U. Protacio [1], Tresor O. Mukiza [1,2], Mari K. Davidson [1], and Wayne P. Wahls [1] *

[1] Department of Biochemistry and Molecular Biology, University of Arkansas for Medical Sciences, 4301 West Markham Street (slot 516), Little Rock, AR 72205-7199, USA

[2] Present address: Department of Cell and Molecular Biology, St. Jude Children's Research Hospital, 262 Danny Thomas Place, Memphis, TN 38105-3678

*To whom correspondence should be addressed. Voice: +1 (501) 686-5787; Fax: +1 (501) 526-7008; Email: wahlswaynep@uams.edu

This file contains:

Table S1. Genotypes of fission yeast strains
Table S2. DNA sequences of PCR primers

Table S1. Genotypes of fission yeast strains

Strain	Genotype [a]
WSP 0004	*h+ ade6-M210*
WSP 0006	*h+ ade6-M375*
WSP 0016	*h- ade6-M210*
WSP 0018	*h- ade6-M26*
WSP 0022	*h- ade6-M375 leu1-32*
WSP 2650	*h+ ade6-M26 atf1D15::ura4F his3-D1 leu1-32 ura4-D18*
WSP 2651	*h- ade6-M210 atf1D15::ura4F his3-D1 leu1-32 ura4-D18*
WSP 2655	*h+ ade6-M26 pcr1D1::his3F his3-D1 leu1-32 ura4-D18*
WSP 2657	*h- ade6-M210 pcr1D1::his3F his3-D1 leu1-32 ura4-D18*
WSP 2659	*h+ ade6-M375 pcr1D1::his3F his3-D1 leu1-32 ura4-D18*
WSP 5814	*h- ade6-M26*
WSP 5815	*h+ ade6-M26*
WSP 5818	*h- ade6-M375*
WSP 6270	*h+ ade6-M210*
WSP 6540	*h+ ade6-4002 ura4-D18*
WSP 6573	*h+ ade6-4099 ura4-D18*
WSP 6577	*h- ade6-4099 ura4-D18*
WSP 6799	*h+ ade6-M26 rst2D::ura4F ura4-D18*
WSP 6806	*h- ade6-M210 rst2D::ura4F ura4-D18*
WSP 6807	*h+ ade6-M210 ura4-D18 leu1-32 rst2D::ura4+*
WSP 6811	*h+ ade6-4002 rst2D::ura4F ura4-D18*
WSP 6815	*h+ ade6-4099 rst2D::ura4F ura4-D18*
WSP 6890	*h+ ade6-M26 php2D::kanMX6*
WSP 7485	*h+ ade6-4002 atf1D15::ura4F his3-D1 ura4-D18*
WSP 7487	*h+ ade6-4099 atf1D15::ura4F his3-D1 ura4-D18*
WSP 7508	*h- ade6-4099 php2D::kanMX6*
WSP 7510	*h+ade6-4002 php2D::kanMX6*
WSP 7519	*h- ade6-m210 php2D::kanMX6*
WSP 7520	*h+ ade6-m210 php2D::kanMX6*
WSP 7531	*h+ ade6-M210 php3D::kanMX6 ura4-D18*
WSP 7537	*h- ade6-4099 php3D::kanMX6*
WSP 7541	*h- ade6-M26 php3D::kanMX6*
WSP 7543	*h- ade6-4002 php3D::kanMX6*
WSP 7553	*h+ ade6-4002 pcr1D1::ura4F ura4-D18*
WSP 7558	*h+ ade6-4099 pcr1D1::ura4F ura4-D18*
WSP 7692	*h- ade6-4099 ura4-D18 leu1-32 rst2D::ura4+*
WSP 7704	*h+ ade6-4002 php5::kanMX6 ura4-D18*
WSP 7713	*h- ade6-M210 php5::kanMX6*
WSP 7715	*h+ ade6-M375 php5::kanMX6 ura4-D18*
WSP 7720	*h+ ade6-M26 php5::kanMX6*
WSP 7722	*h+ ade6-4099 php5::kanMX6 ura4-D18*
WSP 7816	*h- ade6-4002*
WSP 8011	*h+ ade6-M210 ura4-D18 leu1-32 rst2D::ura4+ (pREP3X-Rst2-LEU2)*
WSP 8013	*h- ade6-4099 ura4-D18 leu1-32 rst2D::ura4+ (pREP3X-Rst2-LEU2)*
WSP 8026	*h- ade6-M210 ura4-D18 leu1-32 pcr1D::ura4F*

WSP 8033	*h+ ade6-4099 ura4-D18 leu1-32 pcr1D::ura4F*
WSP 8040	*h+ ade6-4099 ura4-D18 leu1-32 pcr1D::ura4F (pREP3X-Rst2-LEU2)*
WSP 8045	*h- ade6-M210 ura4-D18 leu1-32 pcr1D::ura4F (pREP3X-Rst2-LEU2)*
WSP 8078	*h- ade6-4099 pat1-114 pcr1-HA::kanMX6*
WSP 8082	*h- ade6-4002 pat1-114 pcr1-HA::kanMX6*
WSP 8086	*h- ade6-4099*

[a] Fission yeast nomenclature uses a "D" to designate deletion. These are referred to as "Δ" in the text and figures for the sake of clarity. The *ade6-M26* allele contains the *M26* DNA sequence motif; *ade6-4002* contains the *CCAAT* DNA sequence motif; *ade6-4099* contains the *Oligo-C* DNA sequence motif. See **Table 1** for DNA sequences and locations of all *ade6* alleles.

Table S2. DNA sequences of PCR primers

Amplicon	Primer [a]	Sequence	Purpose [b]
Cam1	*cam1+258F*	5'-GCCTACTGCCGCCGAATTAC-3'	RT-PCR
	cam1+376R	5'-GGCTTCGCGAACTTCCTCTTC-3'	RT-PCR
Php5	*php5+593F*	5'-GTCAAGCTCATATGTCTGCTACT-3'	RT-PCR
	php5+678R	5'-GGCATTGGAAACCCTTGATTG-3'	RT-PCR
Php3	*php3+200F*	5'-CATCTGAACAATGCACTCAAGAA-3'	RT-PCR
	php3+253R	5'-CTCTCTGTATTTGGTGAGAGAAATC-3'	RT-PCR
Php2	*php2+891F*	5'-TGTACTACCAGCGAGCGATA-3'	RT-PCR
	php2+926R	5'-CAATCTCGTACCCTTCAGGAATAG-3'	RT-PCR
Pcr1	*pcr1+126F*	5'-CAATGCTGCCTTTGAGCAATC-3'	RT-PCR
	pcr1+207R	5'-CAACCCTGATGGGCAAGTAAT-3'	RT-PCR
Atf1	*atf1+1037F*	5'-GTACTCAGCAACAGCCTATGT-3'	RT-PCR
	atf1+1131R	5'-CGTGGTTTGGCGTAATGTTAAT-3'	RT-PCR
Rst2	*rst2+844F*	5'-GACGATGAAAGTTTGGCTAGAATG-3'	RT-PCR
	rst2+926R	5'-CAATCTCGTACCCTTCAGGAATAG-3'	RT-PCR
Hsp9	*hsp9-299F*	5'-GCGCTCATTGGAAATAGCTTAC-3'	ChIP
	hsp9-199R	5'-GCTCGCTATCCAATCAGACAA-3'	ChIP
Cdc18	*cdc18+1310F*	5'-GGAAAGCTCAACATGACAACAC-3'	ChIP
	cdc18+1412	5'-GCACTAGCAGATTGGCTCATA-3'	ChIP
Ade6	*ade6+104F*	5'-CAGCAAATTCTCCTGCCAAAC-3'	ChIP
	ade6+190R	5'-GACAGTTCAACAATTGCATCGG-3'	ChIP

[a] The number after each primer name indicates position of the 5' end of the primer relative to the first nucleotide in the start codon of the gene (+1). F, forward primer; R, reverse primer.

b Purpose: RT-PCR, reverse-transcription PCR; ChIP, chromatin immunoprecipitation.
